# Is video‐assisted thoracoscopic lobectomy associated with higher overall costs compared with open surgery? Results of best evidence topic analysis

**DOI:** 10.1111/1759-7714.13708

**Published:** 2021-02-05

**Authors:** Alfonso Fiorelli, Stefano Forte, Francesco Paolo Caronia, Francesco Ferrigno, Mario Santini, René Horsleben Petersen, Wentao Fang

**Affiliations:** ^1^ Department of Translation Medicine, Thoracic Surgery Unit Università della Campania “Luigi Vanvitelli” Naples Italy; ^2^ Istituto Oncologico del Mediterraneo (IOM) Catania Italy; ^3^ Thoracic Surgery Unit Ospedale Civico Palermo Italy; ^4^ Pneumology Unit Ospedale Mauro Scarlato Scafati Italy; ^5^ Department of Cardiothoracic Surgery Copenhagen University Hospital Rigshospitalet Denmark; ^6^ Department of Thoracic Surgery Shanghai Chest Hospital, Jiao Tong University Medical School Shanghai China

**Keywords:** Hospital costs, lobectomy, lung cancer, thoracoscopy, thoracotomy

## Abstract

Thoracoscopic lobectomy has become the preferred approach for surgical management of early stage lung cancer, but the potential higher operative costs limit its widespread use. Theoretically, higher direct costs may be significantly counterbalanced by lower indirect costs, resulting in lower overall costs for thoracoscopic than for open lobectomy. To support this hypothesis, we reviewed the literature until May 2020, analyzing all papers comparing the cost of thoracoscopic versus open lobectomy.A total of 20 studies provided the most applicable evidence to evaluate this issue. In all the studies apart from one, thoracoscopic lobectomy was associated with higher operative costs due to the increased use of disposable instruments, and prolonged operative time. By contrast, in 17 studies the increased operative costs were significantly offset by indirect costs which were lower in thoracoscopic than in open lobectomy due to fewer postoperative complications, faster recovery, and lower readmission rates. It translated into lower overall costs for thoracoscopic than for open lobectomy in 10 studies, similar costs in seven, and higher in three, despite the lower hospitalization costs. The low bed fees and high prices of disposable instruments in these three studies may explain the discordance. The careful use of disposable instruments, and the minimizing hospitalization costs can reduce the total costs of thoracoscopic lobectomy to levels similar or to below those of open lobectomy. The worry that video‐assisted thoracoscopic surgery lobectomy (VATSL) might be associated with an increased overal cost is thus not warranted, and should not be used as an excuse against the use of VATS in surgery for early stage lung cancers.

## Graphical abstract

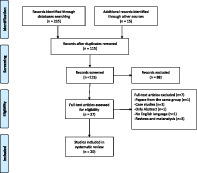

- Is video‐assisted thoracoscopic lobectomy associated with higher overall costs compared with open surgery? A review of the literature until May 2020 was performed, and 20 studies provided the most applicable evidence to evaluate this issue.
- Thoracoscopic lobectomy was associated with higher operative costs than open lobectomy, but lower indirect costs. It translated into lower overall costs for thoracoscopic than for open lobectomy in 10 studies, similar in seven, and higher in three studies.
- The worry that thoracoscopic lobectomy might be associated with increased total cost is thus not warranted, and should not be used as an excuse against the use of thoracoscopy in surgery for early stage lung cancers.

## Clinical scenario

You plan to start a VATS lobectomy (VATSL) program, but the manager of your hospital has some concerns about the cost. He underlines that VATSL may be associated with an increase in hospital costs due to longer operative time, and greater consumption of disposable instruments than open lobectomy (OPENL). You reply that the analysis of VATSL costs should include not only the direct costs related to surgery, but also the other indirect costs related to the length of hospital stay (LOHS), pharmacy and manpower consumption for increased complications, output clinical visits, and readmission. Since VATSL is associated with shorter LOHS and lower postoperative morbidity and mortality, it leads to less health care use after discharge, resulting in lower costs over a longer time horizon than OPENL. You undertake a literature search to support your hypothesis.

## Why this question is important?

Surgery is the only curative treatment for early stage non‐small cell lung cancer (NSCLC), and lobectomy is still the most effective resection.^1^, ^2^ Lobectomy is performed using thoracotomy or VATS. The current guidelines including these from American College of Chest Physicians,^3^ and National Comprehensive Cancer Network^4^ recommend VATSL over OPENL for early stage NSCLC due to less postoperative pain, fewer postoperative morbidity and mortality, and shorter LOHS, and similar oncological results.^5^, ^6^, ^7^, ^8^, ^9^, ^10^, ^11^ However, OPENL is still the most widely used approach. Only approximately 45% of lobectomies registered in the Society of Thoracic Surgeons database were performed thoracoscopically.^12^, ^13^ A survey of European Society of Thoracic Surgeons (ESTS) reported that VATSL was performed by 49% of responders, but only 15% of them used this approach in over 30% of patients with early stage lung cancer.^14^

The higher operative costs for VATSL than for OPENL is one of the main concerns for widespread adoption of VATSL. Although this issue has been evaluated in several studies,^15^, ^16^, ^17^ it is still under debate. Thus, we reviewed the literature analyzing overall health costs of VATSL (direct and indirect costs) to establish whether VATSL is indeed associated with higher hospital costs than OPENL.

## Search strategy

The study design was structured according to the PRISMA protocol.^18^ A literature review was carried out using MEDLINE, PubMed, Scopus, Google Scholar, and Cochrane databases until the end of May 2020 to label all studies comparing VATSL versus OPENL costs. The following MeSH search headings were used: (vats lobectomy.mp. OR VATS LOBECTOMY) AND (thoracotomy.mp. OR THORACOTOMY/) AND (hospital cost.mp. OR HOSPITAL COST/).

Additional papers, abstracts, chapter of books, letters and editorials were retrieved from bibliographies by manual research. The Science Citation Index was used to cross reference for further studies that met the criteria of the study.

## Selection process

Papers were included in the review if they fulfilled the following criteria: (i) papers published in English; (ii) a study population including patients who had undergone VATSL and OPENL; (iii) results comparing costs between OPENL and VATSL. We excluded (i) studies published in languages other than English; (ii) reviews, meta‐analyses, abstracts, case reports and case series; (iii) papers from the same groups. In these cases, only the most recent publication was reported to avoid duplication; (iv) papers comparing only the outcomes, but not the costs between the two procedures.

First, the titles of papers were inspected to decide whether they were appropriate for the purpose of the study. Second, the abstracts of the selected papers were evaluated, and those that were not appropriate were excluded. Third, the remaining articles were thoroughly inspected to decide whether they should be included. Any disagreements were judged by the three senior reviewers (MS, RP and VWF) after referring to the original articles.

**Figure 1:**
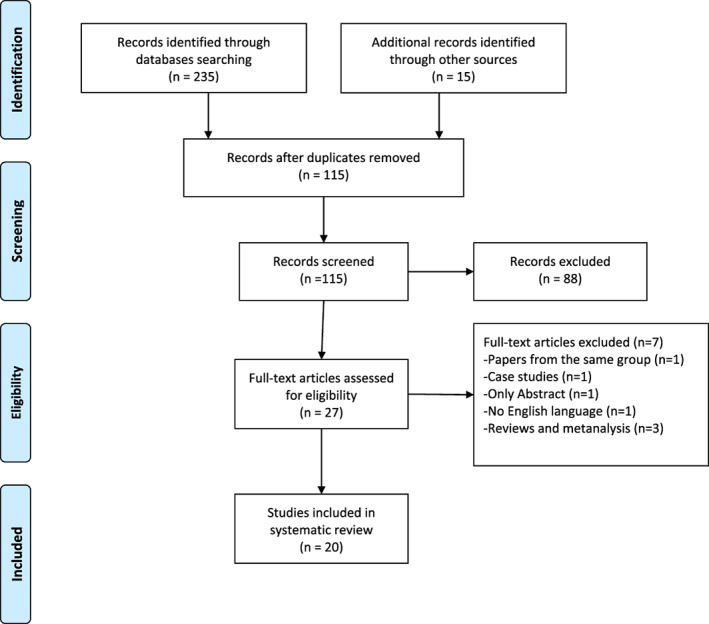
Flow chart of the study according to PRISMA guidelines.^18^

The flow chart of the study is listed in Figure 1. A total of 250 articles were selected using the above reported databases (*n* = 235), and the additional manual (*n* = 15) searches from the references of the selected articles. A total of 135 papers were excluded as being duplicates. Among the 115 papers screened, 88 were excluded based on the titles and abstracts. Of the remaining 27 studies, seven studies were further excluded. Thus, 20 papers were included in the analysis. The authors, year of publication, country, study design, level of evidence based on the criteria of Centre for Evidence Based Medicine,^19^ study population, outcomes, study limitations and conclusions were extracted from the selected papers and are summarized in Table 1.

**Table 1:** Summary of selected papers^20^, ^21^, ^22^, ^23^, ^24^, ^25^, ^26^, ^27^, ^28^, ^29^, ^30^, ^31^, ^32^, ^33^, ^34^, ^35^, ^36^, ^37^, ^38^, ^39^

Authors	Study design and level of evidence	Study population	Outcomes	Results(OPENL vs. VATSL)	Limits	Conclusions
Marijic *et al*.^20^ (2020) Germany	Retrospective analysis of National claims database Level 3a	VATSL: 294 OPENL: 588 Period:2013	**Surgery** ‐LOHS −three‐year survival **Costs (Euros)** ‐ Hospital stay ‐ Hospital care ‐ Output physician care ‐ Drug prescription ‐ Rehabilitation −three‐year lung cancer related costs	11 vs. 9; *P* < 0,001 69.1% vs. 73.8%; *P* = 0.1 12.281 vs. 11.921; *P* = 0.5 18.126 vs. 16.846; *P* = 0.1 2.386 vs. 1.922; *P* = 0.01 3.159 vs. 2.018; *P* = 0.06 58 vs. 57; *P* = 0.9 23.723 vs. 20.828; *P* = 0.02	OPENL included higher number of patients undergoing adjuvant therapy. No information was available on TNM stage or cancer specific survival.	VATSL and OPENL had similar costs
Bendixen *et al*.^21^ (2019) Denmark	RCT Level 1a	OPENL: 99 VATSL: 102 Period: 2008–2014	**Surgery** ‐ Operative time (days) ‐ LOHS (days) **Costs (Euros)** ‐ Medical services ‐ Outpatient clinic ‐ Readmissions ‐ Total cost ‐ QALY	79 (60–101) vs. 100 (80–115); *P* < 0.001 6.7 ± 7.6 vs. 4.8 ± 3.7; *P* < 0.001 6757 ± 4410 vs. 7544 ± 5776; *P* = 0.03 61.575 ± 63.209 vs. 51.412 ± 51 035; *P* = 0.01 51.734 ± 86.456 vs. 29.247 ± 60.548; *P* < 0.001 134.945 ± 120.963 vs. 103.108 ± 90.792; *P* < 0.001 0.830 ± 0.13 vs. 0.851 ± 0.16; *P* = 0.04		VATS was cost‐effective compared to OPENL
Subramanian *et al*.^22^ (2019) Unites States	Retrospective analysis of National claims database Level 3a	OPENL: 8.501 VATSL: 4.608 Period: 2008–2014	**Surgery** ‐ In‐hospital mortality ‐ Major complications ‐ Minor complications ‐ LOHS (days) ‐ Prolonged LOHS (>14 days) ‐ 90‐day readmission **Costs ($)** ‐ Index hospitalization cost ‐ 90‐day costs	2.2% vs. 1.4%; *P* < 0.001 12.1% vs. 7.1%; *P* < 0.001 45.7% vs. 50%; *P* < 0.001 7 (5–9) vs. 5 (4–8); *P* < 0.001 12.1% vs. 7.8%; *P* < 0.001 11% vs. 9.6%; *P* = 0.001 17 200 vs. 17 802; *P* < 0.001 18 464 vs. 18 832; *P* < 0.001	Cancer‐specific data including stage, histology, tumor size, or location were not evaluated.	VATSL was associated with lower costs than OPENL
Kneuertz *et al*.^23^ (2019) United States	Retrospective single center study Level 3b	OPENL: 240 VATSL: 161 Period: 2012–2017	**Surgery** ‐ LOHS (days) ‐ Operative time (minutes) ‐ ICU (days) ‐ Any complications ‐ Major complications ‐ Mortality **Costs ($)** ‐ Total direct ‐ Total indirect ‐ Operating room ‐ Total charges	5.4 vs. 3.8; *P* < 0.001 278 vs. 305; *P* = 0.05 6.8 vs. 5; *P* = 0.1 55% vs. 45%; *P* = 0.1 15% vs. 12%; *P* = 0.7 2% vs. 5%; *P* = 0.1 18 074 vs. 17 259; *P* = 0.6 16 993 vs. 16 414; *P* = 0.7 8697 vs. 9491; *P* = 0.1 120 811 vs. 124 026; *P* = 0.8	Post‐discharge costs were not evaluated	VATSL and OPENL had similar costs
Lipinska *et al*.^24^ (2019) Poland	Retrospective single center study Level‐3b	OPENL: 38 VATSL: 32 Period: 2017	**Surgery** ‐ Operative time (minutes) ‐ LOHS (days) **Costs (Euros)** ‐ Procedure ‐ Hospitalization	143 vs. 145; *P* = 0.9 5.7 vs. 3.6; *P* = 0.0000084 682 vs. 1.705; *P* = 0.01 1.500 vs. 2.235; *P* = 0.05	Small sample size Low bed fees and high disposable instruments costs	VATSL and OPENL had similar costs
Wang *et al*.^25^ (2016) Taiwan	Retrospective analysis of National claims database Level 3a	OPENL: 3.166 VATSL: 2.200 Period: 2004–2010	**Surgery** ‐ Anesthesia time (hours) ‐ LOHS (days) ‐ Tube stay (days) ‐ Surgical mortality **Costs ($)** ‐ Total hospital ‐ Operative ‐ Anesthesia ‐ Nursing ‐ Pharmacy ‐ ICU ‐ Ordinary ward ‐ Laboratory ‐ Treatments ‐ Others ‐ 30‐day after discharge cost	5.5 ± 1.9 vs. 5.4 ± 1.7; *P* = 0.003 17.4 ± 15.8 vs. 13 ± 8.7; *P* = 0.0001 8.6 ± 5.1 vs. 6.4 ± 4.4; *P* = 0.0001 33 (1.04%) vs. 9 (0.41%); *P* = 0.009 6329 ± 4434 vs. 6574 ± 3605; *P* = 0.02 1638.1 ± 310 vs. 1897 ± 362; *P* = 0.0001 548 ± 211 vs. 534 ± 188; *P* = 0.007 614 ± 729 vs. 447 ± 499; *P* < 0.0001 501 ± 1156 vs. 349 ± 1388; *P* < 0.0001 217 ± 401 vs. 144 ± 293; *P* < 0.0001 250 ± 227 vs. 192 ± 122; *P* < 0.0001 893 ± 648 vs. 773 ± 551; *P* < 0.0001 496 ± 788 vs. 358 ± 538; *P* < 0.0001 1169 ± 1094 vs. 876 ± 1033; *P* < 0.0001 831 ± 1759 vs. 612.8 ± 1401; *P* = 0.0001	VATSL presented younger patient with less comorbidity Different hospital‐level	VATSL was associated with higher total hospital costs but lower 30 days after discharge costs than OPENL
Watson *et al*.^26^ (2016) United States	Retrospective analysis of Truven MarketScan Database Level 3a	Lobectomy (VATS = 270; OPEN = 669) Wedge resection (VATS = 1.332; OPEN = 340). Period:2010	**Index of hospitalization** ‐ LOHS (days) ‐ Net hospital payments($) ‐ Key physician payments ($) **Health care utilization (% of increased ratio)** ‐ Office visits ‐ Hospital outpatient visits ‐ ER visits ‐ Inpatient services ‐ Estimated days of health care utilization ‐ Health care expenditure ‐ Drug expenditure	**Average difference; *P*‐value** 1.79; *P* < 0.0001 3496; *P* = 0.009 433; *P* = 0.010 **Postoperative 90‐day/365‐day** 108.5; *P* = 0.27/105.19; *P* = 0.3 123.8; *P* = 0.02/112.93; *P* = 0.1 1.15; *P* = 0.54/1.28; *P* = 0.1 1.86; *P* = 0.008/1.22; *P* = 0.2 127.7; *P* = 0.0002/113.90; *P* = 0.03 124.1; *P* = 0.06/102; *P* = 0.8 134.5; *P* = 0.020/117.70; *P* = 0.22	Economic benefits related to early returning to work was not evaluated	VATS resection was associated with lower hospital costs than OPENL
Deen *et al*.^27^ (2014) United States	Retrospective single center Level 3b	OPENL: 69 VATSL: 58 Period: 2008–2012	**Surgery** ‐ LOHS (days) ‐ Complications ‐ Operative time (minutes) ‐ Additional procedures **Costs ($)** ‐ Overall ‐ Procedure ‐ Operating room ‐ Ward ‐Supplies ‐ Staplers ‐ICU ‐ Respiratory therapy ‐ Laboratory ‐ Pharmacy ‐ Imaging ‐ PT/OT/ST ‐ Others	5.47 vs. 4.75; *P* = 0.1 30% vs. 31%; *P* = 0.9 180 vs. 202; *P* = 0.02 41% vs. 28%; *P* = 0.1 15 036 vs. 13 829; *P* = 0.2 15 036 vs. 13 662; *P* = 0.1 4301 vs. 4520; *P* = 0.2 2610 vs. 2874; *P* = 0.4 2096 vs. 2683; *P* < 0.001 1536 vs. 2033; *P* < 0.001 2540 vs. 1012; *P* = 0.002 1083 vs. 730; *P* = 0.02 935 vs. 563; *P* < 0.001 589 vs. 549; *P* = 0.6 567 vs. 530; *P* = 0.4 178 vs. 146; *P* = 3 131 vs. 52; *P* = 0.3	VATS group had higher number of patients with early stage.	VATSL and OPENL had similar costs
Farjad *et al*.^28^ (2014) United States	Retrospective analysis of MarketScan database Level 3a	OPENL: 6.893 VATSL: 3.069 Period: 2007–2011	**Surgery** ‐ Prolonged LOHS − 90‐day emergency department use − 90‐day readmission **90‐day cost ($)** ‐ Total ‐ Index hospitalization ‐ Readmission ‐ Outpatient health care ‐ Outpatient pharmacy	7.2% vs. 3%; *P* < 0.0001 24% vs. 22%; *P* = 0.005 12% vs.10%; *P* = 0.026 46 470 vs. 42 076; *P* = 0.001 37 673 vs. 35 307; *P* = 0.002 36 845 vs. 35 550; *P* = 0.7 3828 vs. 3530; *P* = 0.04 713 vs. 672; *P* = 0.1	No information was available on cancer, surgeon and hospital‐level characteristics	VATSL was associated with lower 90‐day costs than OPENL
Alpay *et al*.^29^ (2014) Turkey	Retrospective single center Level 3b	OPENL: 49 VATSL: 32 Period: 2007–2009	**Surgery** ‐ Hospital stay (days) **Costs (Euros)** ‐ Disposable instruments ‐ Total hospital cost	10.65 ± 6.57 vs. 7.78 ± 5.11; *P* < 0.05 427 ± 470 vs. 2251 ± 1855; *P* < 0.05 3083 ± 1013 vs. 3970 ± 1873; *P* = 0.002	Low bed fees and high disposable items costs	VATSL was associated with higher hospital costs than OPENL
Piwkowski *et al*.^30^ (2013) Poland	Retrospective single center Level 3b	OPENL: 104 VATSL: 108 Period: 2008–2011.	**Surgery** ‐ Operative time (minutes) ‐ Blood loss (mL) ‐ Complication rates ‐ LOHS (days) ‐ Drainage stay (days) **Costs (Euros)** ‐ Theater ‐ Daily hospital ‐ ICU ‐ Disposable device ***‐*** Total	133 ± 37 vs. 128 ± 35; *P* = 0.2 250 vs. 50; *P* = 0.0001 46% vs. 23%; *P* < 0.0006 10 ± 6.5 vs. 7 ± 3.4; *P* < 0.0012 4,3 *vs*. 3,2; *P* = 0.004 479 vs.1.395; *P* = 0.0001 1.000 vs. 700; *P* = 0.0001 1.000 vs. 930; *P* = 0.03 161 vs. 1.069; *P* = 0.0001 2.047 vs. 2.445; *P* = 0.004	Low bed fees and the high disposable devices costs.	VATS resection was associated with higher hospital costs than OPENL
Ramos *et al*.^31^ (2012) France	Retrospective single center Level 3b	OPENL: 189 VATSL: 98 Period: 2007–2009	**Surgery** ‐ Operative time (minutes) ‐Length of theater stay (days) ‐ LOHS (days) **Costs (Euros)** ‐ Hospital stay ‐ HDU and ICU ‐ Theater and disposable items ‐ Laboratory ‐ Radiology ‐ Total	142 (40.0) vs. 219 (56.5); *P* < 0.001 210 (42.5) vs. 290 (71.3); *P* < 0.001 8 (5.0) vs. 7 (3.0); *P* < 0.001 3.170 vs. 2.502; *P* < 0.001 2.611 vs. 1.929; *P* = 0.1 2.260 vs. 2.861; *P* < 0.001 662 vs. 479; *P* < 0.001 578 vs. 452; *P* = 0.01 14.145 vs. 11.934; *P* < 0.001	Post‐discharge costs were not valuated	VATS resection was associated with lower hospital costs than OPENL
Swanson *et al*.^32^ (2012) United States	Retrospective analysis from Premier Perspective database Level 3a	VATSL: 1.054 OPENL: 2.907 Period: 2007–2008	**Surgery** ‐ LOHS ‐ Surgery time (hours) ‐ Adverse event **Costs ($)** ‐ Hospital ‐ Low vs. high volume surgeons for: ‐ OPEN ‐VATS	7.83 vs. 6.15; *P* < 0.000 3.75 vs. 4.09; *P* < 0.000 lower in VATS (*P* = 0.01) 21 016 vs. 20 316; *P* = 0.02 Similar: 21000. 22 050 vs. 18 133; *P* < 0.05	Cancer‐specific data and post‐discharge costs were not valuated	VATS resection was associated with lower hospital costs than OPENL
Cho *et al*.^33^ (2011) Korea	Retrospective single center Level 3b	VATSL (*n* = 86) OPENL (*n* = 97) Period: 2007–2009	**Surgery** ‐ Complications ‐ Operative time (minutes) ‐ Tube stay (days) ‐ Hospital stay (days) **Costs ($) (all patients and not complicated patients)** ‐ Total hospital ‐ Ward stay ‐ Anesthesia ‐ Surgical material ‐ Surgical fee ‐ Benefit‐service cost ‐ Nonbenefit‐service cost	42 (43.8%) vs. 14 (16.3%); *P* < 0.000 136.4 vs. 145.8; *P* = 0.7 9.1 vs. 5.8; *P* < 0.000 11.9 vs. 7.1; *P* < 0.000 5593 vs. 5391; *P* = 0.09 4769 vs. 4684; *P* = 0.891 429 vs. 268; *P* < 0.000 327 vs. 234; *P* < 0.000 474 vs. 435; *P* = 0.1 478 vs. 455; *P* = 0.3 1365 vs. 1742; *P* < 0.000 1306 vs. 1853; *P* < 0.000 911 vs. 910; *P* = 0.8 900 vs. 91; *P* = 0.7 4119 vs. 3882; *P* = 0.1 3639 vs. 3362; *P* = 0.8 1144 vs. 1305; *P* = 0.6 1130 vs. 1322; *P* = 0.4	No fast‐track discharge protocol was used for OPENL	VATSL and OPENL had similar total hospital costs
Gopaldas *et al*.^34^ 2010 United States	Retrospective analysis of Nationwide Inpatient Sample (NIS) database Level 3a	OPENL: 12.860 VATSL: 759 Period: 2004–2006	**Surgery** ‐ LOS ‐ Complications: ‐ Intraoperative ‐ Overall ‐ Mortality **Costs ($)** ‐ Hospitalization	9.3 ± 0.1 vs. 9.2 ± 0.4; *P* = 0.6 2.8% vs. 4.1%; *P* = 0.03 43.1% vs. 44.1%; *P* = 0.5 3.1% vs. 3.4%; *P* = 0.6 23 862 ± 206 vs. 25 125 ± 1093; *P* = 0.1	The NIS database representing only 20% of all hospital discharges	VATSL and OPENL had similar costs
Burfeind *et al*.^35^ (2010) United States	Retrospective single center Level 3b	OPENL: 37 VATSL: 76 Period: 2002–2004	**Surgery** ‐ Hospital stay (days) ‐ Tube stay (days) **Costs ($)** Total	5 (4–7) vs. 3 (3–4); *P* = 0.0009 3 (3–5) vs. 2 (2–3); *P* = 0.003 12 119 ± 3476 vs. 10 084 ± 2820; *P* = 0.0012	OPENL presented higher patients with advanced stage	VATSL was associated with lower hospital costs than OPENL
Casali *et al*.^36^ (2008) United Kingdom	Retrospective single center Level 3b	VATS (*n* = 93) THOR (*n* = 253) Period: 2004–2006	**Surgery** ‐ Operation time (minutes) ‐ Hospital mortality −30‐day complications ‐ LOHS (days) **Costs (Euros)** ‐ Theater ‐ HDU stay ‐ Ward stay ‐ Total	140 ± 42 vs. 163 ± 34; *P* < 0.0001 2% vs. 1.1%; *P* = 0.4 54.2% vs. 46.2%; *P* = 0.1 6.87 ± 0.19 vs. 5.54 ± 0.37; *P* = 0.001 1.280 ± 54 vs. 2.533 ± 230; *P* = 0.00001 2.571 ± 80 vs. 1.713 ± 236; *P* = 0.00001 4.325 ± 154 vs. 3.776 ± 281; *P* = 0.00001 8.178 ± 167 vs. 8.023 ± 565; *P* = 0.00002	Higher patients with early stage in VATS group	VATS was associated with lower total hospital costs than OPEN
Park *et al*.^37^ (2008) United States	Retrospective single center Level 3b	OPENL: 269 VATSL: 82 Period: 2007	**Surgery** ‐ LOHS (days) ‐Complications (%) **Costs ($)** ‐ Total hospital ‐ Surgeon's fee	6 vs. 5; *P* < 0.001 44% vs. 38%; *P* < 0.001 8368 vs. 1479 515 vs. 0	Cancer‐specific data and post‐discharge costs were not valuated	VATSL was associated with lower total hospital costs than OPENL
Nakajima *et al*.^38^ (1998) Japan	Retrospective single center Level 3b	OPENL: 64/66 VATSL: 8/36 Period: 1997–1998.	**Surgery** ‐ LOS **Costs ($)** ‐ Medications ‐ Laboratory examinations ‐ Total surgical charges ‐ Hospitalization ‐ Total hospital	23.8 ± 7.8 vs. 17.3 ± 7.8; *P* < 0.0001 904 ± 1568 vs. 874 ± 780; *P* > 0.05 1335 ± 632 vs. 990 ± 529; *P* = 0.0064 6174 ± 1383 vs. 5097 ± 747; *P* < 0.0001 3064 ± 1233 vs. 2319 ± 775; *P* = 0.0015 12 178 ± 3877 vs. 9825 ± 2296; *P* = 0.0012	22% of VATS cases underwent lobectomy against 97% of OPEN group	VATS resection was associated with lower hospital costs than OPEN
Sugi *et al*.^39^ (1998) Japan	Retrospective single center Level 3b	OPENL: 20 VATSL: 10 Period: 1992–1995	**Surgery** ‐ LOHS (days) ‐ Operation time (hours) ‐ Chest drainage stay (days) **Costs ($)** ‐ Disposable equipment ‐ Total hospital	27.7 ± 2.4 vs. 25.2 ± 1.7; *P* > 0.05 4.25 ± 0.14 vs. 5.56 ± 0.28; *P* < 0.05 6.3 ± 1.3 vs. 7.1 ± 2.8; *P* > 0,05 457 ± 46 vs. 3660 ± 468; *P* < 0.05 15 052 ± 3751 vs. 18 572 ± 1432; *P* > 0.05	Small cases	VATSL was associated with higher hospital costs than OPENL

ER, emergency room; HDU, high‐dependency unit; ICU, intensive care unit; OPENL, open lobectomy; PT/OT/ST, physiotherapy/occupational therapy/speech therapy; VATSL, video‐assisted thoracoscopy lobectomy.

## Results

Marijic *et al*.^20^ retrospectively compared 882 NSCLC patients who underwent VATSL (*n* = 294) and OPENL (*n* = 588). They found no difference in the hospital stay costs (*P* = 0.5) and hospital care costs (*P* = 0.1) between the two procedures. Compared to OPENL, however, VATSL was associated with lower three‐year lung cancer‐related costs (*P* = 0.02) due to lower outpatient physician care (*P* = 0.01), and drug prescriptions (*P* = 0.06). OPENL included a higher number of patients who underwent adjuvant therapy, which could explain the increased lung cancer related costs in that group.

Bendixen *et al*.^21^ prospectively evaluated 206 patients who underwent VATSL (*n* = 103) and OPENL (*n* = 103). The total costs for VATSL was lower than for OPENL (*P* < 0.001). VATSL was associated with a longer operative time (*P* < 0.001), but shorter LOHS (*P* < 0.001). The post‐discharge costs were lower after VATSL, and this difference was primarily associated with lower costs of readmissions (*P* < 0.001), and outpatient clinics (*P* = 0.012).

Subramanian *et al*.^22^ retrospectively compared 13 109 lung cancer patients who underwent VATSL (*n* = 4608) versus OPENL (*n* = 8501). VATSL compared to OPENL was associated with lower rates of postoperative morbidity (*P* < 0.001) and mortality (*P* < 0.001), shorter LOHS (*P* < 0.001), and fewer 90‐day readmissions (*P* < 0.001), translating into lower index hospitalization cost (*P* < 0.001), and 90‐day cost (*P* < 0.001). After adjusting for patient age, gender, income, comorbidities, and hospital teaching status, VATSL was still less expensive than OPENL.

Kneuertz *et al*.^23^ retrospectively compared 401 patients who underwent OPENL (*n* = 240) versus VATSL (*n* = 161). Procedural cost in the operating room was lower for OPENL, as it was associated with the shortest operating room times (*P* = 0.05) and the least expensive equipment. However, VATSL compared to OPENL was associated with shorter LOHS (*P* < 0.001) and fewer costly events, and, as a result, in similar overall hospital cost (*P* = 0.6). These results were confirmed even after carefully adjusting for patient selection.

Lipinska *et al*.^24^ retrospectively compared 70 patients who underwent OPENL (*n* = 38) versus VATSL (*n* = 32). VATSL was associated with higher operative costs (*P* = 0.01) mainly driven by staplers, but lower LHOS (*P* = 0.000008). The lower hospitalization costs and the high cost of staplers might explain the higher total hospital costs for VATSL than for OPENL (*P* = 0.05).

Wang *et al*.^25^ retrospectively compared 5366 patients who underwent VATSL (*n* = 2.200) or OPENL (*n* = 3.166). VATSL compared to OPENL was associated with higher operative costs (*P* = 0.0001), but this difference was not significantly balanced by lower costs related to anesthesia (*P* = 0.007), ordinary ward (*P* < 0.0001), ICU (*P* < 0.0001), nursing (*P* < 0.0001), and pharmacy (*P* < 0.0001), and hospitalization, resulting into higher total costs for VATSL than for OPENL (*P* = 0.02). By contrast, 30‐day post discharge costs were lower for VATSL than for OPENL (*P* = 0.0001).

Watson *et al*.^26^ retrospectively compared 2611 patients who underwent lobectomy (VATS: 270; OPEN: 669) or wedge resection (VATS: 1332; OPEN: 340). OPEN compared to VATS resections (lobectomy or wedge) were associated with longer LOHS (*P* < 0.0001), and higher payment to hospitals (*P* = 0.009), and physicians (*P* = 0.01). OPENL had 1.28‐times and 1.14‐times more health care utilization days within 90‐day (*P* = 0.0002), and 365 day (*P* = 0.03), respectively, after the operation compared with VATSL, translating into increased expenditures of $3260 at 90 days, and $822 at 365 days for OPEN procedures. No significant differences in utilization were noted between OPEN and VATS wedge resections, except for fewer outpatient visits within 90 days in the OPEN group.

Deen *et al*.^27^ retrospectively compared 127 patients who underwent OPENL (*n* = 69) versus VATSL (*n* = 58) for early stage lung cancer. Complication rates (*P* = 0.94) and LOHS (*P* = 0.11) were similar between the two groups. Operative costs related to operative time (*P* = 0.02) and disposable instrument costs (*P* < 0.001) were higher for VATSL than for OPENL, but this difference was offset by lower ICU costs (*P* = 0.002) and lower laboratory costs (*P* < 0.001), resulting in similar overall costs between the two procedures (*P* = 0.2).

Farajad *et al*.^28^ retrospectively compared 9962 patients who underwent OPENL (*n* = 6893) or VATSL (*n* = 3069). VATSL compared to OPENL was associated with significantly lower total unadjusted 90‐day (*P* = 0.001), index hospitalization (*P* = 0.002), and outpatient use (*P* = 0.04) costs. After adjusting costs for age, sex, comorbidity index, health plan, and use of epidural anesthesia, 90‐day costs were $3476 lower for VATS lobectomy than for OPENL (*P* < 0.001). VATSL was associated with a lower rate of patients with prolonged LOHS (>14 days) than OPENL (*P* < 0.0001), explaining the difference in total costs between the two groups. In fact, adding prolonged LOHS as a covariate to the regression model reduced the differential cost by 63% (−$1276), and the difference between VATSL versus OPENL was no longer significant. In the fully adjusted model, PLOS was associated with the highest cost differential (+$50 820; *P* < 0.001).

Alpay *et al*.^29^ retrospectively compared 81 patients who underwent VATSL (*n* = 32) and OPENL (*n* = 49). LOHS in the VATSL group was significantly shorter than for OPENL (*P* < 0.05), but VATSL was associated with higher costs of disposable surgical instruments (*P* < 0.05). More expensive disposable surgical instruments and cheaper hospital stay charges lead to higher overall costs in VATSL than in OPENL group (*P* = 0.002).

Piwkowski *et al*.^30^ retrospectively evaluated the data of 212 patients who underwent VATSL (*n* = 108) or OPENL (*n* = 104). VATSL was associated with shorter LOHS (*P* < 0.0012), lower complication rate (*P* < 0.0006) and ICU admission rate (*P* < 0.0027), but higher theater costs (*P* = 0.0001) due to increased utilization of staplers (*P* = 0.0001). The significantly higher hospital costs and ICU costs after OPENL did not compensate for the higher theater costs of VATSL, translating into higher total hospital costs for VATSL than for OPENL (*P* = 0.004).

Ramos *et al*.^31^ retrospectively compared the costs of 287 patients who underwent VATSL or segmentectomy (*n* = 98) versus OPENL (*n* = 189). VATSL compared to OPENL was associated with increased intraoperative costs (*P* < 0.0001) due to increased use of disposable surgical instruments and staplers (*P* < 0.001), longer operative time (*P* < 0.001). In VATSL, upper‐right lobectomy and segmentectomy were more expensive than other types of resection. However, the increased surgical costs of VATSL were offset by the lower hospital stay (*P* < 0.001), laboratory (*P* < 0.001), and radiology (*P* = 0.01) costs, resulting in lower overall cost for VATSL than for OPENL (*P* < 0.0001).

Swanson *et al*.^32^ retrospectively compared the costs of 3961 patients who underwent VATSL (*n* = 1054) versus OPENL (*n* = 2907). VATSL was associated with longer operative time (*P* < 0.000), but lower LOHS (*P* < 0.000) and lower risk of adverse events (*P* < 0.019), resulting in lower total hospital costs than OPENL (*P* = 0.02). These differences persisted even after adjusting for patient and hospital characteristics. Only for VATSL the economic impact was magnified as the surgeon's experience increased (*P* < 0.05).

Cho *et al*.^33^ retrospectively compared 183 patients who underwent VATSL (*n* = 86) versus OPENL (*n* = 97) for lung cancer. VATSL compared to OPENL was associated with lower postoperative morbidity (*P* < 0.000); lower chest tube duration (*P* = 0.000); and LOHS (*P* = 0.000), but higher surgical material costs (*P* < 0.000). Cost comparisons were then adjusted for postoperative complications, type of lobectomy, and surgeon's experience. No significant difference was found between VATSL and OPENL among all patients (*P* = 0.09), and among only noncomplicated patients (*P* = 0.8). The overall cost for the VATSL was lower than for the OPENL in cases of right lower lobectomy, left upper lobectomy, and left lower lobectomy, while only the cost of anesthesia was affected by surgeon's experience, being higher for the early than for the experienced period (*P* = 0.009).

Gopalds *et al*.^34^ retrospectively compared 13 619 discharge records of patients who underwent OPENL (*n* = 12 860) or VATSL (*n* = 759). VATSL was associated with a higher risk of intraoperative complications than OPENL (*P* = 0.04), but no differences were found regarding postoperative mortality (*P* = 0.6), complications (*P* = 0.5), and LOHS (*P* = 0.8), resulting in similar overall hospitalization costs (*P* = 0.1). A higher percentage of patients with an annual income >$59 000 underwent VATS lobectomy than patients with an income <$59 000 (*P* < 0.0001). Theoretically, patients of higher socioeconomic status choose VATS because it implicates smaller incisions, and allows the patient to return to work earlier.

Burfeind *et al*.^35^ retrospectively compared 113 patients who underwent OPENL (*n* = 37) versus VATSL (*n* = 76). Total costs were significantly greater for OPENL than for VATSL (*P* = 0.0012), with overall savings of approximately $2000 per patient. The costs were less for TL at all phases of patient care, and the most dramatic savings were in the preoperative phase where OPENL was almost twice as expensive as VATSL. One explanation could be that fewer preoperative tests of patient fitness were performed due to the perceived minimally surgical trauma related to VATS. There was also more surgical staging performed in the prelobectomy setting within the OPENL, perhaps reflecting the surgeon's wishes to confirm the absence of mediastinal involvement before performing a thoracotomy. Even after adjusting for lung cancer stage, total costs for OPENL were still higher than for VATSL (*P* = 0.005).

Casali *et al*.^36^ retrospectively compared 346 patients who underwent VATSL (*n* = 93) versus OPENL (*n* = 253) for stage I or II lung cancer. Total costs for VATSL were lower than for OPENL (*P* = 0.00002). Despite theater room costs being twice as high for VATSL over OPENL (*P* = 0.00001), this difference was significantly offset by reduced costs related to HDU (*P* = 0.00001) and ward‐bed stays (*P* = 0.00001). The operating costs varied according to the type of resection. Among VATS resections, upper bilobectomy was associated with the highest theater cost, €1400 more than left lower lobectomy, which was the least expensive. In this case, the reduced postoperative costs were not able to offset the intraoperative costs. Upper lobectomies and right lower lobectomies were associated with the highest intraoperative cost differences between VATSL versus OPENL, ranging between €2000 and €2500.

Park *et al*.^37^ retrospectively compared 269 patients who underwent OPENL (*n* = 269) versus VATSL (*n* = 82). OPENL was associated with higher LOHS (*P* < 0.001) and complication rate (*P* < 0.001) compared to VATSL, resulting in $5098 of additional cost.

Nakajima *et al*.^38^ retrospectively compared 102 patients with a mixture of primary lung cancer and metastatic disease, 66 of whom had OPEN resection and 36 VATS resection. VATS was associated with lower costs of laboratory examinations (*P* = 0.0064), anesthesia (*P* > 0.05), disposable devices (*P* < 0.0001), and LOHS (*P* = 0.0015). Thus, the total hospital costs for VATS surgery were lower than for OPEN resection (*P* = 0.0012). However, 64 of 66 OPEN patients underwent lobectomy, whereas only eight of 36 VATS patients had lobectomy. This difference undoubtedly favored the VATS group and explained the decreased costs and hospital stay in that group.

Sugi *et al*.^39^ retrospectively compared 30 patients who underwent VATSL (*n* = 10) versus OPENL (*n* = 20). VATSL compared to OPENL was associated with higher overall costs (*P* > 0.05) due to higher disposable costs (*P* < 0.05), and longer operative time (*P* < 0.05) and similar LOHS (*P* > 0.05).

## Discussion

The present analysis included 19 retrospective cohort studies,^20^, ^22^, ^23^, ^24^, ^25^, ^26^, ^27^, ^28^, ^29^, ^30^, ^31^, ^32^, ^33^, ^34^, ^35^, ^36^, ^37^, ^38^, ^39^ and one randomized controlled trial (RCT).^21^ One study presented a level of evidence of 1a being a RCT,^21^ seven a level of evidence 3a due to retrospective multicenter design,^20^, ^22^, ^25^, ^26^, ^28^, ^32^, ^34^ and 12 a level of evidence 3b being retrospective single center studies.^23^, ^24^, ^27^, ^29^, ^30^, ^31^, ^33^, ^35^, ^36^, ^37^, ^38^, ^39^ All studies^20^, ^21^, ^22^, ^23^, ^24^, ^25^, ^26^, ^27^, ^28^, ^29^, ^30^, ^31^, ^32^, ^33^, ^34^, ^35^, ^36^, ^37^, ^38^, ^39^ evaluated the direct costs (ie, operation time and disposable instruments), and the indirect hospitalization costs (ie, ICU stay, LOHS, postoperative examinations, and surgical outcomes); additionally, six studies^20^, ^21^, ^22^, ^25^, ^26^ also evaluated the health costs after‐discharge (ie, outpatient clinic visit, rehabilitation, pharmacy use, and readmission).

### Direct costs of VATSL versus OPENL

In all studies^20^, ^21^, ^22^, ^23^, ^24^, ^25^, ^26^, ^27^, ^28^, ^29^, ^30^, ^31^, ^32^, ^33^, ^34^, ^36^, ^37^, ^38^, ^39^ apart from one,^35^ VATSL was associated with significantly higher operative costs compared to OPENL due to the increased use of disposable instruments, and prolonged operative time. During thoracotomy, vessels are usually ligated by sutures, while staplers are reserved for closing the bronchus and dividing the fissure, but, during VATSL, vessels and bronchus are all closed with staplers, in addition to the fissures. Additional costs are driven by the use of energy devices for hilar dissection and lymph node resection. Three studies^31^, ^33^, ^36^ also stratified the operative costs for different types of lobectomy, and found that upper lobectomy was more expensive than other types of lobectomy due to the different need for reloads. An upper lobectomy required a mean of three reloads to divide the pulmonary artery branches, while a lower lobectomy needed just one reload most of the time. Yet, two studies^32^, ^33^ stratified the operative costs for the surgeon's experience. One study^32^ found that the increased level of surgeon experience was associated with a reduction of operative time, while another study^33^ found no difference as experienced surgeons could volunteer to take more difficult cases that required longer procedures. Only one study^35^ reported lower operative costs for VATSL than for OPENL. The results could be explained by the fact that the authors used the same endostaplers during VATS and thoracotomy.

### Indirect costs of VATSL versus OPENL

In 17 studies^20^, ^21^, ^22^, ^23^, ^24^, ^25^, ^26^, ^27^, ^28^, ^31^, ^32^, ^33^, ^34^, ^35^, ^36^, ^37^, ^38^ the indirect costs for VATSL was lower than for OPENL due to fewer postoperative complications, faster recovery, shorter ICU stay and LHOS, and lower output patient visits and readmission rates. Thus, the lower indirect costs significantly balanced the higher operative costs, resulting in lower overall costs for VATSL than for OPENL in 10 studies,^21^, ^22^, ^26^, ^28^, ^31^, ^32^, ^35^, ^36^, ^37^, ^38^ or in similar overall costs in seven studies.^20^, ^23^, ^24^, ^25^, ^27^, ^33^, ^34^ In only three studies,^29^, ^30^, ^39^ the significantly higher hospital costs of OPENL did not compensate for higher operative costs of VATSL, translating into higher total hospital costs for VATSL than for OPENL. Two of these studies came from Turkey and Poland^29^, ^30^ where the low bed fees and high prices for disposable instruments could explain this discordance.

In this analysis, the assessment of the comparative costs for VATSL and OPENL was limited by several issues. All papers^20^, ^22^, ^23^, ^24^, ^25^, ^26^, ^27^, ^28^, ^29^, ^30^, ^31^, ^32^, ^33^, ^34^, ^35^, ^36^, ^37^, ^38^, ^39^ but one^21^ were retrospective with consequent intergroup differences due to selection bias. VATSL patients were frequently younger, with earlier stage lung cancer, and lower preoperative comorbidity,^20^, ^22^, ^23^, ^28^, ^32^, ^35^, ^36^ but the cost comparison was adjusted for these factors in only four studies. On the other hand, in five studies^20^, ^22^, ^28^, ^32^, ^37^ cancer‐specific data including stage, histology, tumor size, or location were not evaluated as the referred database did not contain these specific data. Thus, it is difficult to discuss the impact of these differences on cost as those factors could be responsible for more morbidity, prolonged stay, and higher hospital‐stay related cost of the OPENL group. Other limitations were the different medical and social conditions in various countries, the difference in operative techniques used for both VATSL and OPENL, or the different anatomy that could make the operation difficult, and the inability of current cost models to capture aspects of quality of life after operation.

In conclusion, the current evidence showed that VATSL was associated with higher operative costs than OPENL, but lower indirect costs during and after discharge. This translated into lower or similar overall costs for VATSL than for OPENL in most of the studies. Thus, the careful use of disposable instruments, and minimizing the health costs during and after discharge can reduce the overall costs of VATSL to levels similar or below those of OPENL. The worry that VATSL might be associated with increased total cost is thus not warranted, and should not be used as an excuse against the use of VATS in surgery for early stage lung cancers.

## Disclosure

Alfonso Fiorelli, Stefano Forte, Francesco Paolo Caronia, Francesco Ferrigno, Mario Santini and Wentao Fang disclose no conflict of interest and no funding for the present paper. René Horsleben Petersen is speaker fee from Medtronic.
